# Soccer Game Turned Bloody: A Case of Exercise-Induced Ischemic Colitis

**DOI:** 10.14309/crj.0000000000001406

**Published:** 2024-06-21

**Authors:** Duha Zaffar, Eliesther Rivera, Stephen Schwartz, Osman Ali, Bruce D. Greenwald

**Affiliations:** 1 Department of Internal Medicine, University of Maryland Medical Center Midtown, Baltimore, MD; 2 American University of Antigua, St John's, Antigua and Barbuda; 3 Division of Gastroenterology and Hepatology, Department of Medicine, University of Maryland School of Medicine, Baltimore, MD

**Keywords:** Ischemic colitis, gastrointestinal bleeding, physical activity, sports

## Abstract

Ischemic colitis (IC) should be considered as a cause for gastrointestinal symptoms in patients with recent vigorous physical activity. Vasoconstriction driven by increased sympathetic tone during exercise is believed to mediate exercise-induced IC. In this report, a 21-year-old man with no medical history developed self-resolving, sudden-onset hematochezia and abdominal pain after playing in a collegiate soccer match for 90 minutes. Colonoscopy with biopsy showed changes consistent with IC. He improved without further treatment. In most cases, exercise-induced IC resolves completely with supportive care and correction of hypovolemia. Careful monitoring is appropriate before pursuing further evaluation.

## INTRODUCTION

Exercise-induced ischemic colitis (IC) is not uncommon. Gastrointestinal (GI) symptoms including abdominal cramps, diarrhea, and bloody stools are reported in 20%–50% of endurance athletes, more often in runners.^1^ In 1 study, 2% of marathon runners reported blood in stool after races, and 20% of runners had positive fecal occult blood test after marathons.^2^ The aim of this case report is to heighten awareness of IC as a possible cause for hematochezia and abdominal pain in those with recent vigorous sports activity.

## CASE REPORT

**Figure 1.**
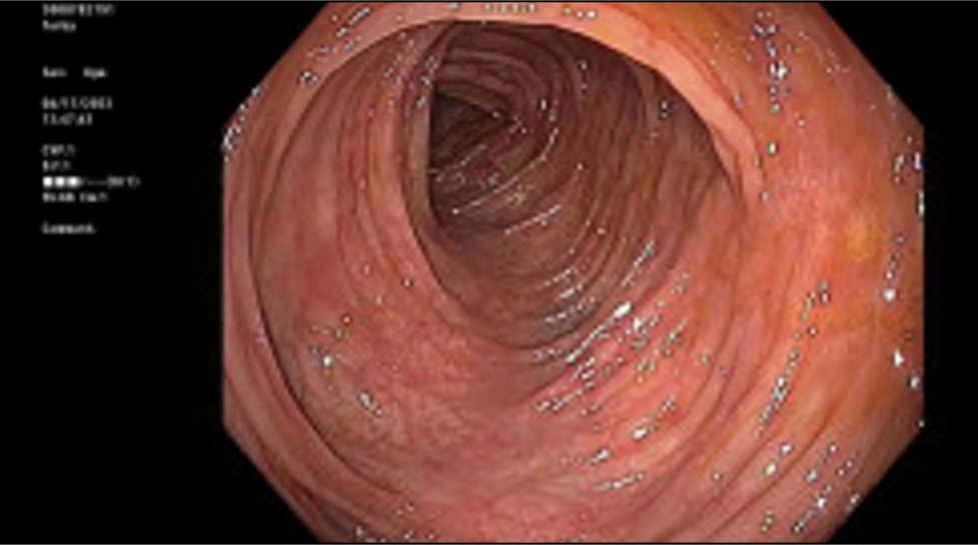
Patchy areas of granular and pale mucosa in the distal transverse colon.

**Figure 2.**
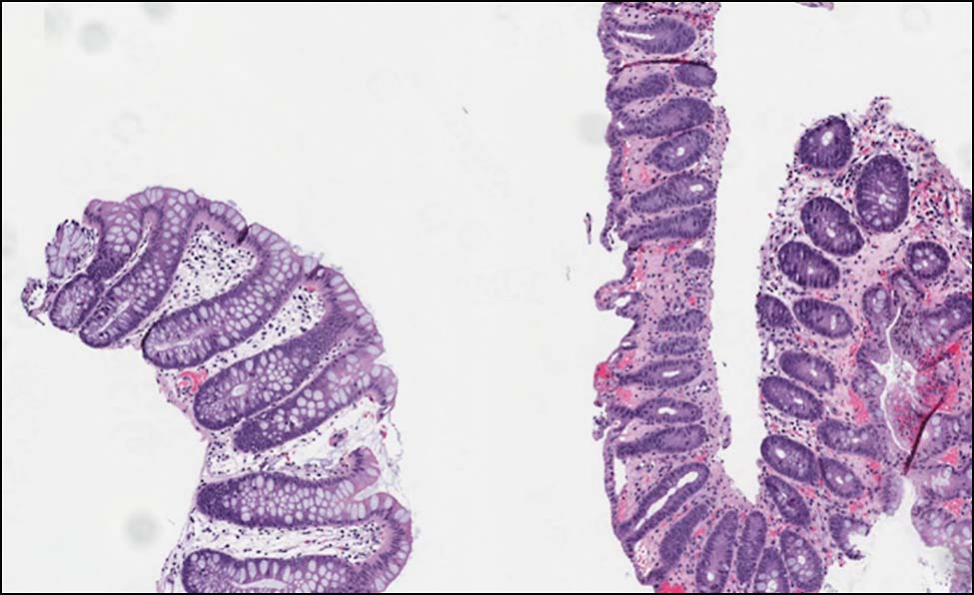
Biopsy of the transverse colon; on the left is the normal colon, and on the right is patient's colon showing ischemic changes with crypt injury and lamina propria hyalinization. Magnification: 200×.

The patient is a 21-year-old male college student with no medical history who presented with abdominal pain, hematochezia, and coffee ground emesis after playing nearly the entire 90 minutes of a National Collegiate Athletics Association Division 1 soccer match. The patient did not have a history of hematochezia and denied use of nonsteroidal anti-inflammatory drugs, performance-enhancing medications, or illicit substances. On admission, bradycardia and abdominal discomfort were noted. Laboratory test results revealed elevated serum creatinine (2.1 mg/dL) consistent with acute kidney injury, elevated creatinine kinase (504 U/L), and lactic acidosis (3.1 mmol/L). Abdominal/pelvic computed tomography angiography was normal. Mild gastropathy was noted on upper GI endoscopy. Colonoscopy revealed ischemic colopathy in the splenic flexure, transverse colon, hepatic flexure, and ascending colon (Figure 1). Biopsies from the ascending and transverse colon showed crypt injury and lamina propria hyalinization (Figure 2) consistent with ischemic changes in the colon. He was managed with intravenous fluids, and all symptoms resolved within 24 hours. He was counseled to avoid strenuous physical activity for 2 weeks and to maintain careful hydration before, during, and after exercise to maintain normal blood pressure for intestinal perfusion.

## DISCUSSION

There is a significant rise in body's metabolic demands during endurance athletics, such as long-distance running in a marathon. This rise in metabolic demand triggers an adaptative response involving release of epinephrine and norepinephrine. These neurotransmitters increase sympathetic tone, leading to an increase in the cardiac output. Simultaneously, the blood flow is diverted toward the more metabolically active tissues, skeletal muscles, and the heart, which require a higher blood supply during vigorous exercise. Consequently, when sympathetic output peaks, blood supply to the mesentery is diminished, often by 50%–80% compared with the supply at rest.^3^ This reduction becomes more pronounced with more strenuous and prolonged exercise.^4^ Hypovolemia is another potential complication experienced during endurance training, primarily resulting from increased water loss through sweating, not adequately balanced by increasing oral fluid intake. Athletic runners are notably more susceptible to these issues, particularly when compared with people engaged in various other sports disciplines. Elite athletes carry a significantly higher risk in contrast to those who engage in running solely as a recreational activity.^1^ The increased risk and incidence of GI symptoms during strenuous physical activity pose a substantial impediment for athletes as it limits their performance.

The colon represents the second most common location for exercise-induced GI bleeding.^5^ Anatomical variations exist in the colon blood supply, which increases the risk of IC in some individuals and can lead to variations in clinical presentation.^6^ Watershed areas, defined as critical terminal arterial supply areas where vascular territories converge, are more prone to ischemia. The splenic flexure and sigmoid colon are particularly at high risk, given their limited blood supply. Right-sided IC has been shown to carry a higher mortality risk when compared with left-sided cases,^7^ although left-sided cases are more common in clinical practice.^8^

Commonly reported symptoms of exercise-induced IC include sudden onset of crampy abdominal pain, sometimes out-of-proportion to physical examination. Patients can also experience bloody stools, bloating, abdominal cramps, diarrhea, fecal incontinence, heartburn, nausea, vomiting, chest pain, and urge to defecate.^9^ Most of the cases of exercise-induced IC are mild. These milder forms often go undetected because they tend to be transient and self-limiting, thus not requiring further diagnostic procedures. In cases of diagnostic uncertainty, colonoscopy is the gold standard diagnostic tool because it has shown to be the most sensitive test for colonic ischemia.^6^ A range of findings are observed during colonoscopy, including hyperemia, petechia, erosions, mucosal friability and bleeding, strictures, and ulcerations.^6,8,10^ Pathology evaluation of histologic manifestations demonstrates accumulation of neutrophils and other inflammatory cells, edema, necrosis, thrombosis, crypt loss, and hemorrhage.^6,8,10^ It is essential to acknowledge that the more transient or milder cases may not show microscopic or histologic changes compared with more severe or prolonged ones.

Strategies for preventing IC include conditioning with gradual increase in duration and intensity of exercise, limiting exercise intensity, avoiding training in excessive heat, proper hydration before, during, and after exercise, and judicious nonsteroidal anti-inflammatory drug use.^5,11^ Our patient's symptoms resolved within 24 hours, and management consisted of intravenous fluids and observation. Short-term abstention from exercise was also recommended to allow for proper healing of the colon, although no guidelines exist to guide duration. In a broader context of health management and disease prevention in athletes, addressing risk factors that could predispose them to IC becomes essential to optimize their peak performance and prevent health decline in those engaged in rigorous exercise.

## DISCLOSURES

Author contributions: D. Zaffar wrote the manuscript and reviewed the literature. E. Rivera also contributed to the manuscript writing and literature review. D. Zaffar, S. Schwartz, O. Ali, and B. Greenwald reviewed and edited the manuscript and are the article guarantors.

Financial disclosure: O. Ali is supported by NIH NIDDK T32 DK067872-19 grant.

Previous presentation: This case report was presented at the ACG Annual Conference; October 24, 2023; Vancouver, Canada.

Informed consent was obtained for this case report.

## References

[1] de Oliveira EP Burini RC. The impact of physical exercise on the gastrointestinal tract. Curr Opin Clin Nutr Metab Care 2009;12(5):533–8. 19535976 10.1097/MCO.0b013e32832e6776

[2] Eichner ER. Gastrointestinal bleeding in athletes. Phys Sportsmed 1989;17(5):128–40. 10.1080/00913847.1989.11709788 27447272

[3] Rowell LB. Human cardiovascular adjustments to exercise and thermal stress. Physiol Rev 1974;54(1):75–159. 4587247 10.1152/physrev.1974.54.1.75

[4] Joyner MJ Casey DP. Regulation of increased blood flow (hyperemia) to muscles during exercise: A hierarchy of competing physiological needs. Physiol Rev 2015;95(2):549–601. 25834232 10.1152/physrev.00035.2013 PMC4551211

[5] Moses FM. Exercise-associated intestinal ischemia. Curr Sports Med Rep 2005;4(2):91–5. 15763045 10.1097/01.csmr.0000306079.74945.ea

[6] Baixauli J Kiran RP Delaney CP. Investigation and management of ischemic colitis. Cleve Clin J Med 2003;70(11):920-1. 14650467 10.3949/ccjm.70.11.920

[7] Brandt LJ Feuerstadt P Blaszka MC. Anatomic patterns, patient characteristics, and clinical outcomes in ischemic colitis: A study of 313 cases supported by histology. Am J Gastroenterol 2010;105(10):2245–53. 20531399 10.1038/ajg.2010.217

[8] Brandt LJ Feuerstadt P Longstreth GF Boley SJ. ACG clinical guideline: Epidemiology, risk factors, patterns of presentation, diagnosis, and management of colon ischemia (CI). Am J Gastroenterol. 2015;110(1):18–45. 25559486 10.1038/ajg.2014.395

[9] Cohen DC Winstanley A Engledow A Windsor AC Skipworth JR. Marathon-induced ischemic colitis: Why running is not always good for you. Am J Emerg Med 2009;27(2):255.e5–7. 10.1016/j.ajem.2008.06.033 19371557

[10] Cubiella Fernandez J Nunez Calvo L Gonzalez Vazquez E . Risk factors associated with the development of ischemic colitis. World J Gastroenterol 2010;16(36):4564–9. 20857527 10.3748/wjg.v16.i36.4564 PMC2945488

[11] Papantoniou K Michailides C Bali M Papantoniou P Thomopoulos K. Gastrointestinal bleeding in athletes. Ann Gastroenterol 2023;36(3):267–74. 37144023 10.20524/aog.2023.0788 PMC10152804

